# A novel clinical metaproteomics workflow enables bioinformatic analysis of host-microbe dynamics in disease

**DOI:** 10.1128/msphere.00793-23

**Published:** 2024-05-23

**Authors:** Katherine Do, Subina Mehta, Reid Wagner, Dechen Bhuming, Andrew T. Rajczewski, Amy P. N. Skubitz, James E. Johnson, Timothy J. Griffin, Pratik D. Jagtap

**Affiliations:** 1 Department of Biochemistry, Molecular Biology and Biophysics, University of Minnesota, Minneapolis, Minnesota, USA; 2 Minnesota Supercomputing Institute, University of Minnesota, Minneapolis, Minnesota, USA; 3 Department of Laboratory Medicine and Pathology, University of Minnesota, Minneapolis, Minnesota, USA; University of Saskatchewan, Saskatoon, Saskatchewan, Canada

**Keywords:** microbiome, metaproteomics, clinical analysis, bioinformatics

## Abstract

Clinical metaproteomics has the potential to offer insights into the host-microbiome interactions underlying diseases. However, the field faces challenges in characterizing microbial proteins found in clinical samples, usually present at low abundance relative to the host proteins. As a solution, we have developed an integrated workflow coupling mass spectrometry-based analysis with customized bioinformatic identification, quantification, and prioritization of microbial proteins, enabling targeted assay development to investigate host-microbe dynamics in disease. The bioinformatics tools are implemented in the Galaxy ecosystem, offering the development and dissemination of complex bioinformatic workflows. The modular workflow integrates MetaNovo (to generate a reduced protein database), SearchGUI/PeptideShaker and MaxQuant [to generate peptide-spectral matches (PSMs) and quantification], PepQuery2 (to verify the quality of PSMs), Unipept (for taxonomic and functional annotation), and MSstatsTMT (for statistical analysis). We have utilized this workflow in diverse clinical samples, from the characterization of nasopharyngeal swab samples to bronchoalveolar lavage fluid. Here, we demonstrate its effectiveness via analysis of residual fluid from cervical swabs. The complete workflow, including training data and documentation, is available via the Galaxy Training Network, empowering non-expert researchers to utilize these powerful tools in their clinical studies.

**IMPORTANCE:**

Clinical metaproteomics has immense potential to offer functional insights into the microbiome and its contributions to human disease. However, there are numerous challenges in the metaproteomic analysis of clinical samples, including handling of very large protein sequence databases for sensitive and accurate peptide and protein identification from mass spectrometry data, as well as taxonomic and functional annotation of quantified peptides and proteins to enable interpretation of results. To address these challenges, we have developed a novel clinical metaproteomics workflow that provides customized bioinformatic identification, verification, quantification, and taxonomic and functional annotation. This bioinformatic workflow is implemented in the Galaxy ecosystem and has been used to characterize diverse clinical sample types, such as nasopharyngeal swabs and bronchoalveolar lavage fluid. Here, we demonstrate its effectiveness and availability for use by the research community via analysis of residual fluid from cervical swabs.

## INTRODUCTION

Mass spectrometry (MS)-based metaproteomics enables the analysis of proteins expressed by microbial communities and can be applied to clinical samples to understand microorganism contributions to disease (1). Metaproteomics provides insight into how the microbiome responds to a diseased condition by direct characterization of functional molecules (proteins) that are beyond the capabilities of metagenomics approaches, which mainly focus on taxonomic characterization (2). Moreover, clinical metaproteomics can provide insights into how the microbiome interacts with its host environment. However, one current challenge of metaproteomic analysis of clinical samples is that the high relative abundance of host (human) proteins can hamper the detection and identification of lower abundance microbial proteins. Moreover, identifying microbial peptides derived from tryptic digestion of isolated proteins involves searching tandem mass spectrometry (MS/MS) spectra against large sequence databases comprising all microbial proteomes present in the sample, decreasing sensitivity and increasing the potential for false positives (3). In addition, assigning taxonomy to the detected peptide sequences presents challenges due to the conservation of protein sequences across taxa (4). Assigning functions to detected proteins can also present challenges mainly due to a lack of confident annotation of encoded proteins (4, 5). Here, we offer a novel bioinformatics workflow that overcomes many of these challenges and enables effective metaproteomic analysis in clinical samples relevant to studies of disease.

To demonstrate the effectiveness of our clinical metaproteomics workflow, here we analyze selected MS/MS data from Pap test fluid (PTF) samples collected from ovarian cancer (OC) and non-OC patients. The bioinformatics workflow is accessible via the Galaxy ecosystem, which offers access to powerful bioinformatic tools for metaproteomic data analysis that facilitate the development and execution of complex workflows necessary for complete clinical metaproteomics (6). Galaxy is a free, browser-based, scalable platform that is maintained by a thriving community to meet emerging needs in bioinformatics analysis across omic domains (7, 8). New users can also access online and on-demand training resources, such as step-by-step instructions, access to workflows, and example data sets via the Galaxy Training Network (GTN) resources (6, 9). We envision these collective bioinformatic resources will find use in many clinical studies, such as potential secondary infections inherent to human infectious diseases and other broad research questions regarding host-microbe interactions underlying human disease and cancer (10, 11). Such clinical metaproteomic studies offer the discovery of new microbe-host responses and interactions, as well as the potential to define peptide targets of interest for the development of targeted MS-based clinical assays for diagnostics and health monitoring.

## RESULTS

### Database generation module: MetaNovo

**Fig 1:**
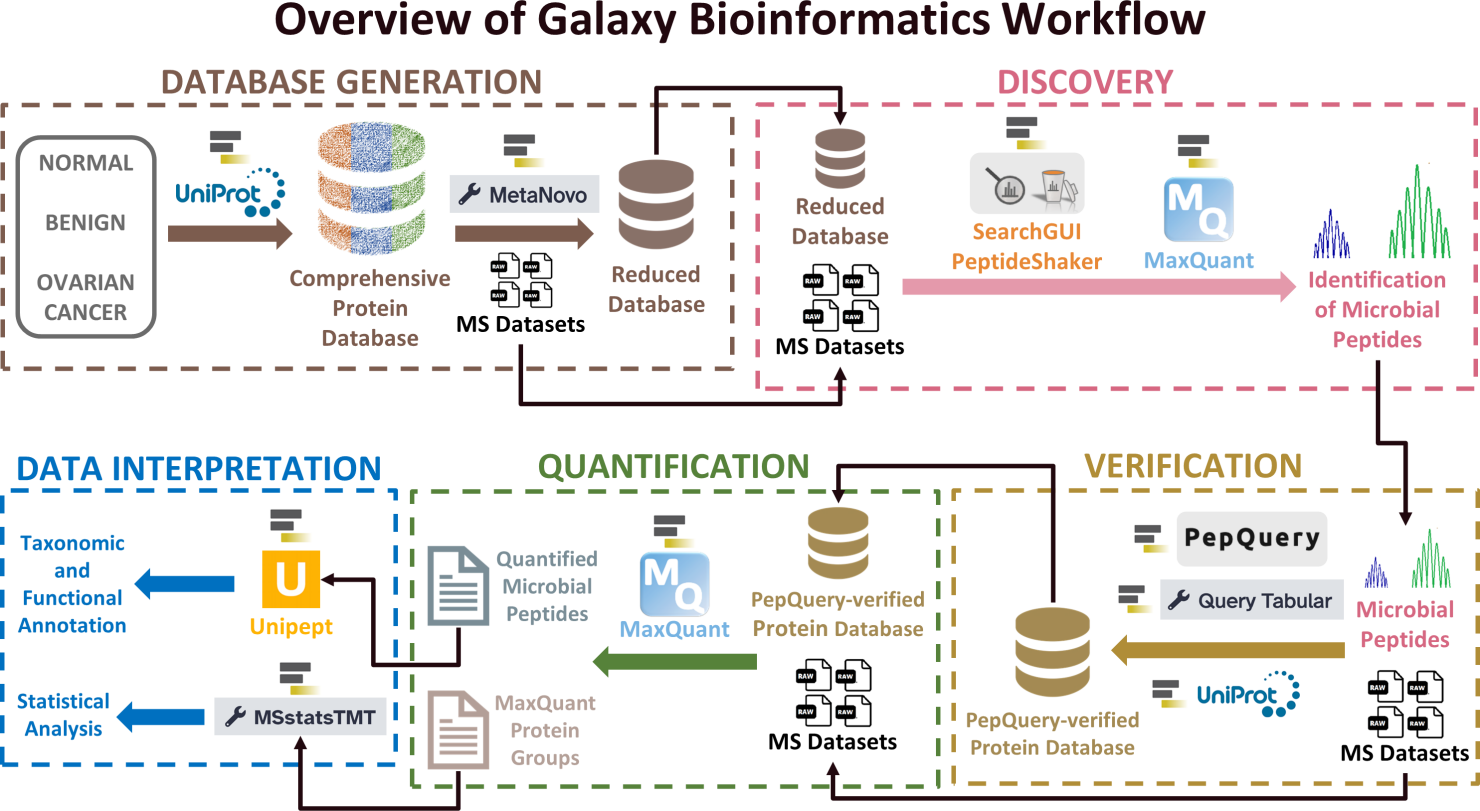
Overview of Galaxy bioinformatics workflow. This figure summarizes the workflow into five modules: Database Generation, Discovery, Verification, Quantification, and Data Interpretation.

**Fig 2:**
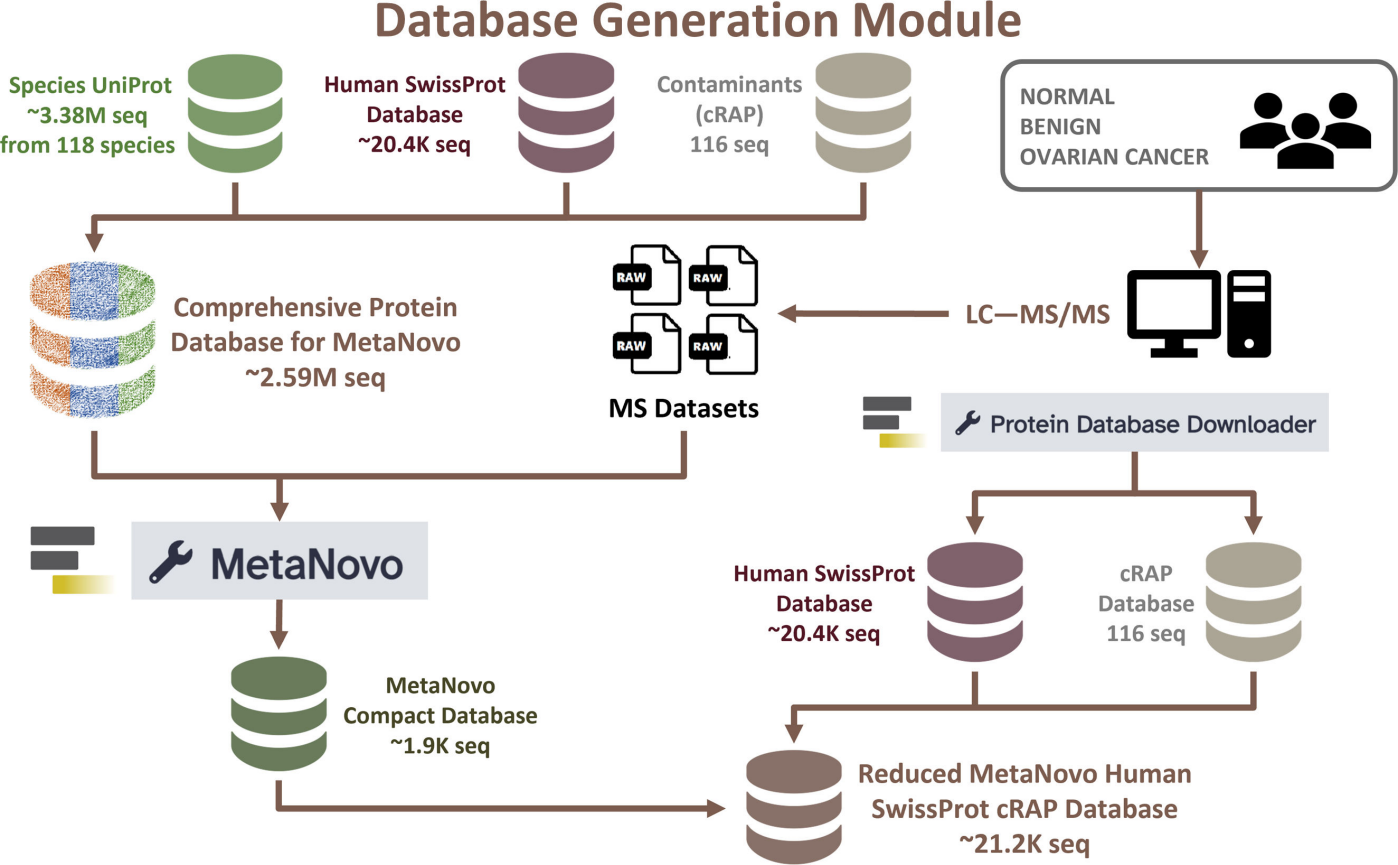
Database Generation module. Overview of a large comprehensive database for input into MetaNovo and reduced database generation using MetaNovo.

All modules described in this workflow are depicted in Fig. 1 and summarized in Table S1, including inputs, software tools, and outputs. The first module of the clinical metaproteomics workflow is database generation, wherein a large database comprising 3,383,217 protein sequences (generated from 118 species) was used (Fig. 2). This database was generated using the UniProt XML Downloader tool. This Galaxy tool can also generate a database from proteomes collected at the genus, family, order, or any other higher taxonomic clade, and from any type of microorganism (e.g., bacteria, virus, fungus, etc.). In order to generate a reduced protein sequence database from this large starting database that will be compatible with standard sequence database searching tools, the MetaNovo tool was used (12, 13). As an input, four example Mascot generic format (MGF) files were processed using the DirecTag tool within MetaNovo to generate a reduced database of 1,908 protein sequences. This reduced database was merged with the Human SwissProt database and contaminants database to generate a database with 21,289 protein sequences so that it could be used for the Discovery module.

### Discovery module: SearchGUI/PeptideShaker and MaxQuant

**Fig 3:**
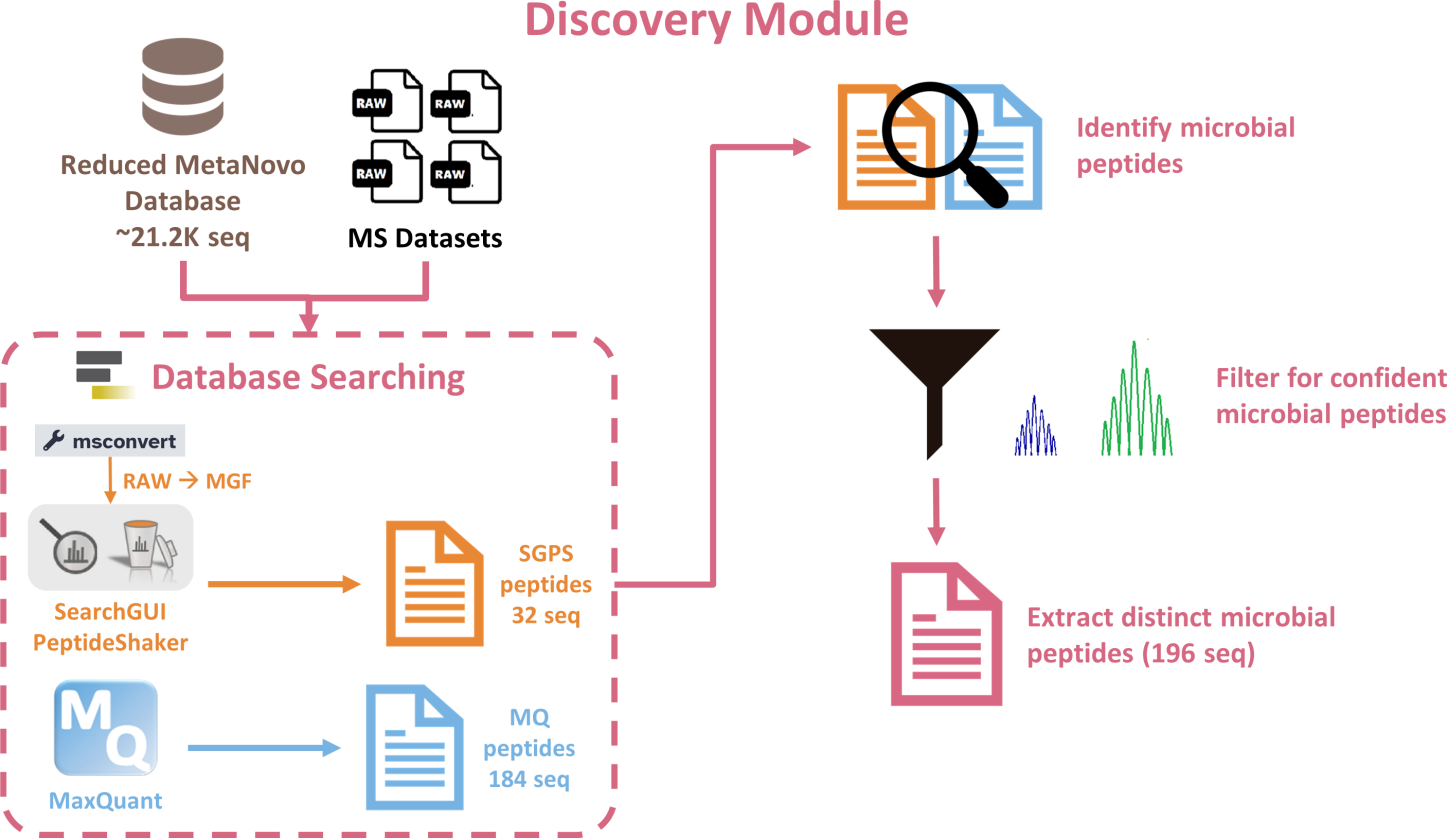
Discovery module. Microbial peptide identification using SearchGUI/PeptideShaker and MaxQuant.

The Discovery module uses the four example RAW files, an experimental design file (for MaxQuant), and MetaNovo protein sequence database (generated from the Database Generation module) (Fig. 3). Using the msconvert tool, MS/MS data (RAW files) were converted to MGF files to search against the MetaNovo-generated database using the SearchGUI/PeptideShaker tool suite and MaxQuant (14–18). The peptides identified in this module were used for the subsequent Verification module. For our example data set, 184 microbial peptides were detected using MaxQuant, and 32 microbial peptides were detected using SearchGUI/PeptideShaker. Peptides from both tools were merged, filtered, and grouped to retain distinct peptides, and as a result, 196 unique microbial peptides were identified.

### Verification module: PepQuery2

**Fig 4:**
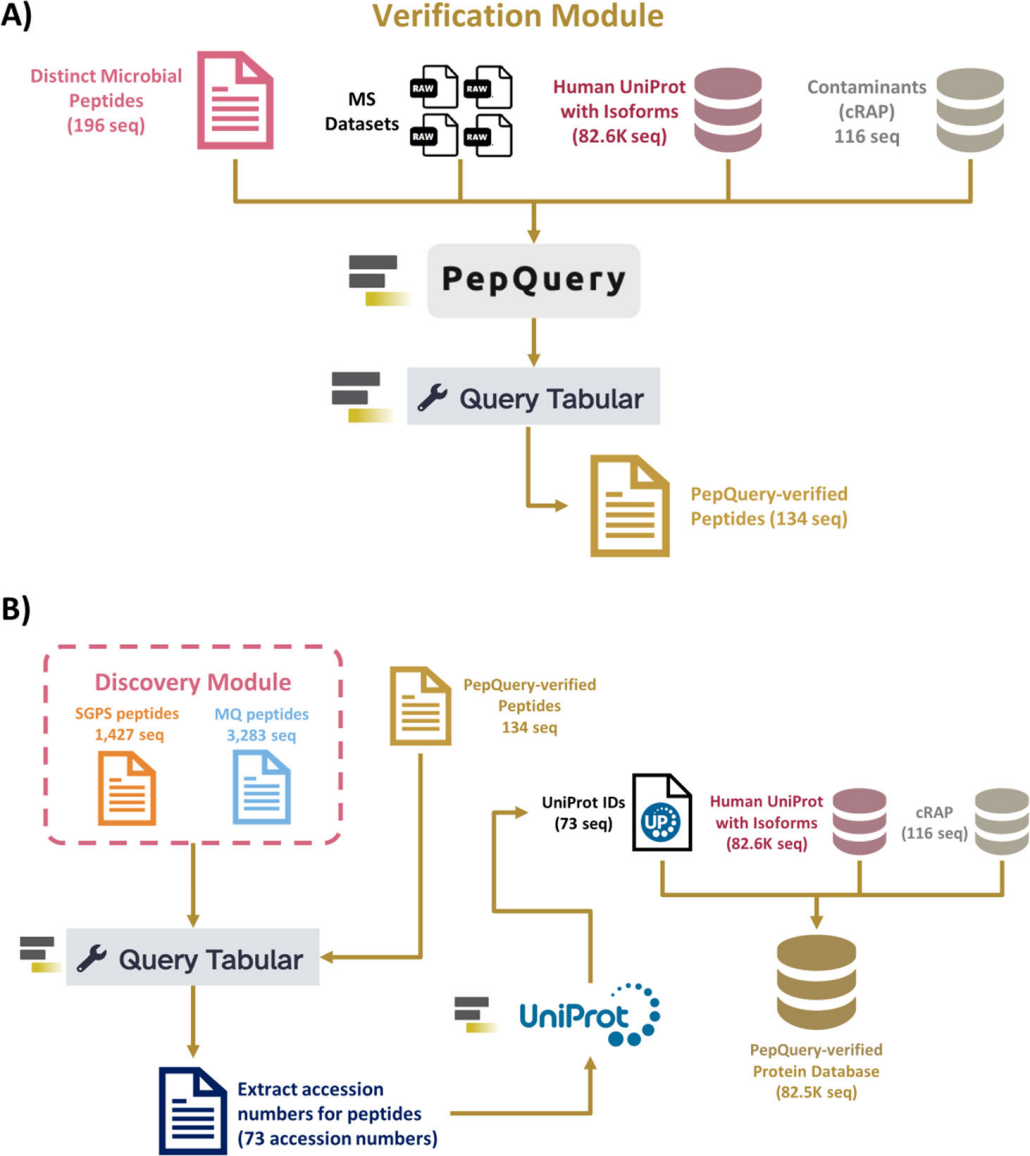
Verification module. Overview of peptide verification using PepQuery that consists of (A) generation of PepQuery-verified peptides, which will be used to (B) generate a PepQuery-verified protein database.

For verification, 196 microbial peptides from the Discovery module, MGF files, and peptide reports generated from MaxQuant and SearchGUI/PeptideShaker were used as inputs. The module uses the PepQuery2 tool to further evaluate MS/MS evidence for candidate microbial sequences identified in the Discovery module (19, 20). The PepQuery2 tool generated 164 confident PSMs, which were used to select 134 microbial peptides that passed the verification criteria for PepQuery2. The Query Tabular tool extracted 73 accession numbers of proteins associated with these 134 microbial peptides verified by PepQuery2 (Fig. 4A) (21). The 73 accession numbers associated with these verified microbial peptides were used to generate a verified microbial protein sequence database for the Quantification module (Fig. 4B).

### Quantification module: MaxQuant

**Fig 5:**
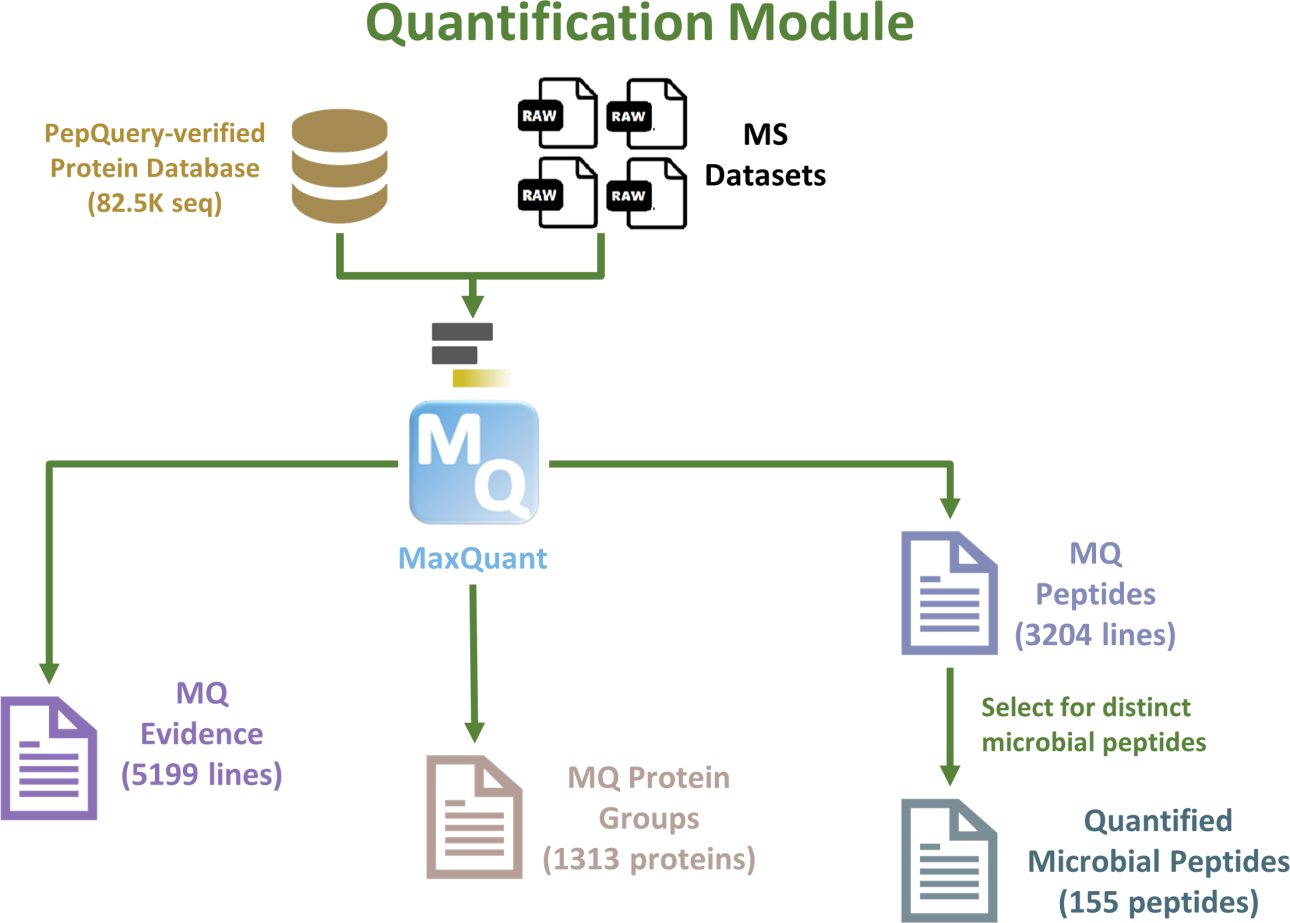
Quantification module. Overview of peptide quantification using MaxQuant.

**Fig 6:**
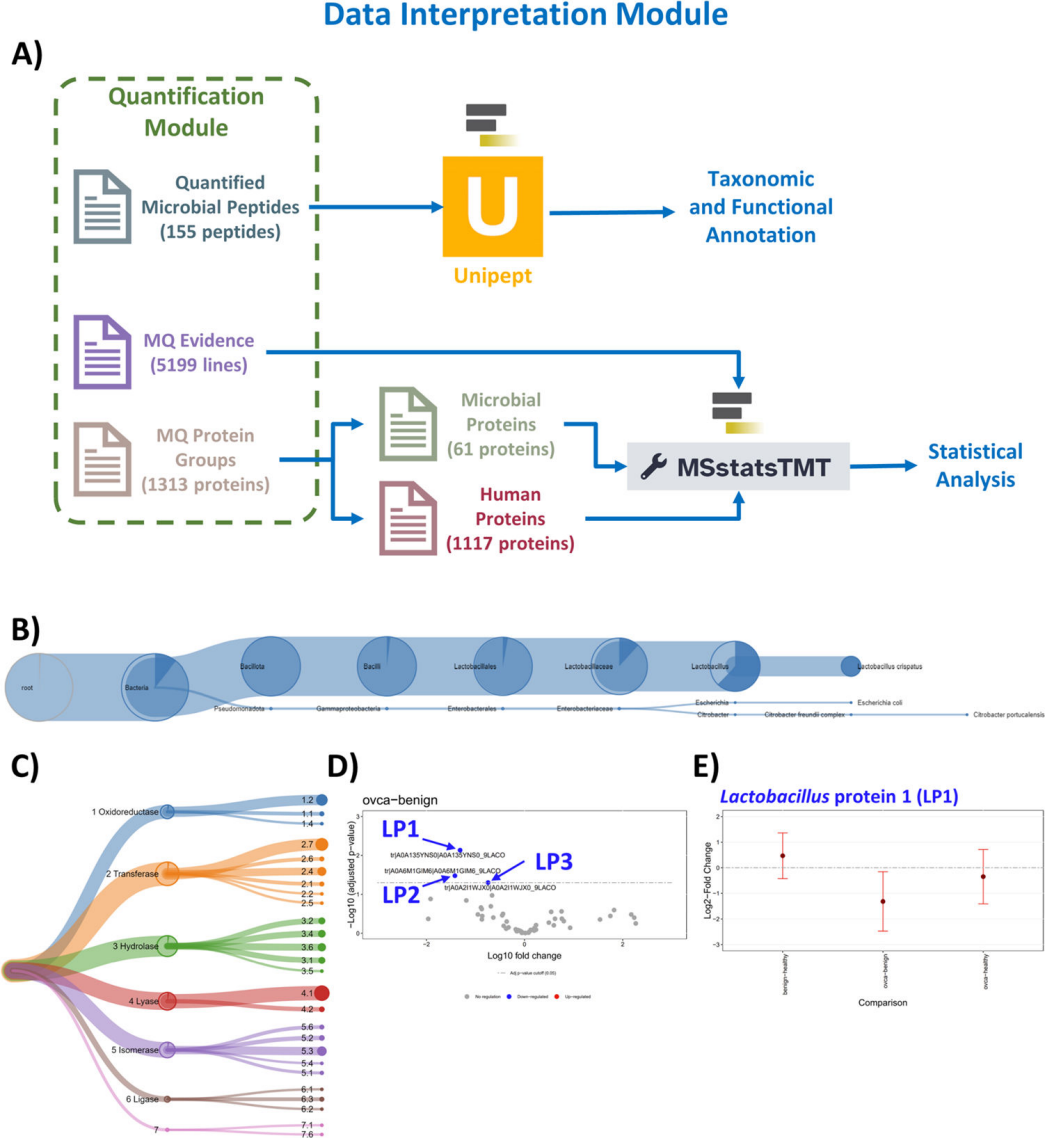
Data interpretation module. Overview of data interpretation module using (**A**) Unipept and MSstatsTMT. Examples of the microbial outputs: (**B**) microbial taxonomy tree, (**C**) microbial enzyme commission proteins tree, (**D**) microbial proteins volcano plot (gray denotes no regulation, red/blue for up-/down-regulated), and (**E**) a comparison plot for microbial protein LP1.

Using the microbial protein sequences containing the verified peptides from PepQuery2, a “compact” database was constructed by merging these with human protein sequences and known contaminants. The MS data sets were searched against this compact database using MaxQuant to generate a peptide report file (3,203 peptides), which was filtered to retain distinct microbial peptides, resulting in a total of 155 quantified microbial peptides (Fig. 5) (16, 17, 22). MaxQuant also identified a total of 1,313 protein groups, which included 1,178 non-contaminant protein groups (1,117 human and 61 microbial; Fig. 5 and 6A). A protein group from MaxQuant contains one or more related protein isoforms that share identified peptide sequences.

### Data interpretation module: Unipept and MSstatsTMT

Unipept provided taxonomic and functional annotation for the 155 quantified microbial peptides (Fig. 6A) (23, 24). The taxonomy tree depicted the likely taxonomic assignments for each peptide sequence (Fig. 6B). *Lactobacillus* was the most abundant genus detected with 90 sequences assigned (56 sequences assigned at genus level and 34 sequences assigned to *L. crispatus*), followed by *Citrobacter* (one sequence assigned to *C. portucalensis*) and *Escherichia* (one sequence assigned to *E. coli*). Unipept also generated an enzyme commission (EC) proteins tree that displays the numerical classification of what chemical reaction is catalyzed. The most prominent classification was transferase with 33 sequences, followed by hydrolase with 29 sequences, and lyase with 24 sequences (Fig. 6C).

The Protein Group text file obtained through MaxQuant quantification contained 1,313 protein groups. After selecting human and microbial protein groups, this resulted in 1,117 human and 61 microbial proteins (Fig. 6A). Additionally, MSstatsTMT generated volcano plots (Fig. 6D) and comparisons (OC, healthy, benign) to identify differentially expressed microbial proteins (Fig. 6E; Fig. S1 and S2). The tandem-mass tag (TMT) module in MSstats was used for quantification as this selected data set was labeled with the TMT reagents for quantitative proteomics (22, 25, 26). As shown in Fig. 6D, a volcano plot visualizes the log-fold changes and negative log10 of adjusted *P*-values for a comparison between OC and benign cases for the 61 quantified microbial proteins. The horizontal dashed line represents the false discovery rate (FDR) cutoff (where adjusted *P*-value = 0.05), and data points above this line denote statistically significant proteins. The data points are colored to denote any differential abundance between cases: gray for no regulation and red/blue for up-/down-regulation. In this study, three microbial proteins from *Lactobacillus* were determined to be statistically significant when comparing OC versus benign cases. For demonstration purposes, these proteins have been labeled *Lactobacillus* protein (LP)1, LP2, and LP3 in Fig. 6D. As shown in Fig. 6E, a comparison plot visualizes log2-fold changes and variation of multiple comparisons for a single protein. An example of one microbial protein (LP1) that was statistically significant when comparing the OC cases to the benign cases is shown in Fig. 6E.

## DISCUSSION

MS-based clinical metaproteomics, which offers insights into the functional molecules expressed by microorganisms as well as taxonomic composition within human samples, has been gaining attention in recent years (1, 11, 27). Moreover, the approach offers insights into how the host environment might interact with microbial communities, via simultaneous analysis of the host proteome. Clinical metaproteomics has been used in unraveling pathogenic mechanisms in Alzheimer’s disease, autism, colorectal cancer, cystic fibrosis, diabetes, inflammatory bowel disease, and COVID-19 co-infection in a variety of clinical sample types including feces, bronchoalveolar lavage fluid, vaginal swabs, and oral cavity specimens (10, 28–40).

This study was meant to demonstrate the effectiveness of our Galaxy-based workflow for clinical metaproteomics, operating on a relatively small example set of quantitative proteomics data from an ongoing study of OC in fluid from cervical swabs. Even though limited, this demonstration data set did identify a protein from the emerging pathogen *Citrobacter portucalensis*. First isolated from an aquatic sample in Portugal, *C. portucalensis* has been detected in blood and fecal samples, and in recent years, it has been identified as an emerging global multi-drug resistant pathogen, stemming from its acquisition of clinically relevant resistance determinants (41–46). The confirmation of the presence of *C. portucalensis* in cervical swabs and its pathogenic potential will require further validation but demonstrates the potential of this bioinformatics pipeline to enable discoveries.

Despite its immense potential in offering functional insights into the microbiome as well as the host response to infection, implementing clinical metaproteomics as a research approach still faces challenges. These include creating and analyzing large protein sequence databases and linking them to the necessary tools for confident peptide and protein identification and quantification, functional and taxonomy annotation, and statistical analysis. Ideally, all these steps would be encapsulated in workflows made publicly available and accessible to the bench researcher (1). Foremost among these challenges is the relatively low abundance of microbial proteins as compared to the host proteins in clinical samples, thus making it difficult to detect and characterize these microbial proteins (10, 31). Although our study does not address sample preparation or instrumental analysis methods that may increase MS detection of microbial proteins, our workflow does employ bioinformatics tools aimed at ensuring the most sensitive and confident identification of MS-detected peptides. Additionally, this bioinformatics workflow simultaneously identifies and quantifies human proteins found within these samples at no additional computational cost (Fig. S3).

Our workflow addresses the fundamental challenge of very large protein sequence databases inherent to MS-based metaproteomics. We used the MetaNovo tool to generate a reduced database from an initial database containing millions of sequences (12). Alternative searching approaches to MetaNovo include the sectioning method and the two-step database methods (47–49). MetaNovo uses *de novo* sequence tag matching along with an algorithm for probabilistic optimization of the large input database to generate a customized, reduced-size protein sequence database (12, 13).

It should also be noted that our workflow does not require prior knowledge of sample microorganism composition or the use of metagenomic information (12). MetaNovo is capable of matching MS/MS data to any FASTA database that is generated either from a review of the literature to estimate microorganisms most likely present in a sample, or from collective protein sequence databases of organisms identified from 16S rRNA or metagenomic sequencing data of the sample. MetaNovo simultaneously identifies proteins from any organism type that are included in the database, including human, bacterial, fungal, viral, or any other organism type. The ability of MetaNovo to operate on large and unbiased sequence databases also avoids the potential for false positives when constraining the size of the database to only selected organisms (12).

The workflow also prioritizes rigorous analysis to generate confident matches to microbial peptides and proteins, which are oftentimes found at lower abundance than human host proteins in clinical samples. This analysis comprises several steps: (i) the Discovery module: the use of two peptide identification programs (SearchGUI/PeptideShaker and MaxQuant) increases the range of detected peptides and results in the detection of multiple microbial peptides; (ii) the Verification module: only microbial peptides that pass the PepQuery2 tool’s verification thresholds are retained; and (iii) the Quantification module: only microbial proteins from verified peptides are used to build the compact database for final peptide and protein identification and quantification.

In the Discovery module, to enhance initial identification of microbial peptides we have used multiple complementary database search algorithms for PSM generation. In this demonstration, we used X!Tandem and MS-GF+ within SearchGUI, which also offers more search algorithms to use if desired (14, 15). The Galaxy implementation of our workflow offers flexibility to use other Galaxy-deployed search algorithms, including emerging methods such as FragPipe and Scribe, that could further enhance advanced metaproteomic and related multi-omic analysis (9, 50, 51).

One step that is often overlooked when using MS-based metaproteomics is the need to ascertain the quality of the PSMs for microbial peptide identification and protein inference. This is especially important since most of the modern search algorithms rely on FDR analysis for the identification of peptides and proteins from data-dependent-acquisition MS data (52). This might lead to false positive PSMs, especially from microbes of low abundance that are identified using large protein sequence databases, which can erroneously bolster the number and quality of PSMs. Therefore, there is a need to verify these proteins using bioinformatic approaches which can lead to further validation via targeted proteomic methods (53, 54). We have described methods such as Peptide-Spectral-Match-Evaluation along with BLAST-P verification and PSM visualization using the Multi-omics Visualization Platform within the Galaxy platform (7, 55, 56). For this workflow, we have used the PepQuery2 tool which verifies putative peptide identifications using peptide-centric database searching (20). This rigorous algorithm evaluates the evidence for MS/MS spectra that support the presence of peptides of interest. The PepQuery2 tool compares other sequences within the human reference and microbial protein sequences in the reduced databases against the queried peptide sequences to ensure that the putatively identified sequence is indeed the best match to the MS/MS spectra within the raw data. The Verification module helps to further reduce the database size, enabling quantitative analysis.

For the Quantification module, we have used MaxQuant software within Galaxy which generates quantitative protein and peptide outputs ready for MSstatsTMT analysis in the Data Interpretation module (22, 25, 26). Although MaxQuant was used in this study, newer software such as FragPipe and Scribe are planned for Galaxy implementation and could also be used for quantitative analysis (50, 51). The Data Interpretation module uses MSstatsTMT and Unipept which enable data interpretation via visualization (23–25). We used the MSstatsTMT tool for this demonstration data set, as it was TMT labeled; however, the MSstats package itself is compatible with label-free quantitative proteomics data as well. We have described the use of variations of this interpretation module for prior clinical metaproteomics studies (57).

The workflow we describe here is built with an eye toward flexibility to incorporate emerging methods and technologies that may further improve clinical metaproteomic studies. We believe that the outputs from the modules described above will aid in prioritizing proteins and peptides of the highest interest. The workflow outputs can be used to provide the necessary information needed to develop targeted assays for validation and possible implementation within the clinic. For example, methods for enriching microbial proteins from clinical samples containing high-abundance host proteins would enhance the depth of detection via MS as well as provide improved starting data, immediately compatible with our workflow. Metaproteomics researchers have also started using data-independent acquisition for quantitative studies, with growing evidence indicating that this approach can greatly improve the depth and quantitative accuracy of measurements (37, 58, 59). Along with the sensitive mass spectrometers available now for deep quantification studies, this offers an opportunity for metaproteomics researchers to go deeper into the microbiome functions along with deciphering taxonomic contributions (38, 60).

Implementation of our described workflows in Galaxy offers a number of benefits, including scalability for handling large data sets and compute-intensive analyses, as well as the aforementioned flexibility for incorporating new software as they emerge (8, 22, 55, 57, 60, 61). Perhaps the biggest advantage of Galaxy is the training resources it offers. Comprehensive training remains a requirement to promote the adoption of advanced bioinformatics tools (6). The GTN (https://training.galaxyproject.org/) was created to provide learners and instructors with free online training materials and access to globally-maintained resources while promoting open data analysis practices (6, 62). The Galaxy platform empowers learners and researchers worldwide, regardless of expertise, with the tools and skills to perform their own data analyses, all readily accessible through a standard web browser.

In summary, we have developed a clinical metaproteomics workflow within the Galaxy platform. This accessible workflow offers researchers all necessary modules for success in analyzing their metaproteomics data, including generating customized protein sequence databases, matching MS/MS data to these sequences using multiple sequence database search algorithms, verifying the quality of spectral matches, quantifying peptides and inferred proteins, and interpreting the taxonomically and functionally annotated data. We anticipate that the availability of this workflow and the underlying software tools will enable metaproteomics researchers to undertake challenging clinical metaproteomics investigations and make important new discoveries about human health.

## MATERIALS AND METHODS

### Sample processing to generate MS/MS spectra

For this workflow development, four example RAW files were used as input MS data sets. These RAW files were a subset of a total of 40 PTF samples from 20 OC and 20 non-OC patients. De-identified residual Pap test sample vials were obtained from the University of Minnesota BioNet Tissue Procurement Facility with approval from the University of Minnesota Institutional Review Board (Protocol 1112M07362), which does not require patient consent for de-identified clinical specimen use. All procedures followed the University of Minnesota Institutional Review Board guidelines and regulations. Clinical specimens were collected from the ectocervix of women undergoing routine cervical cancer screening. Pap tests were processed, stained, and examined by a pathologist for diagnosis, as previously described (63).

Proteins from each sample were isolated, digested into peptides with trypsin, and labeled with a distinct Tandem Mass Tag-11-plex tagging reagent. Each experimental group included one pooled reference sample labeled with a unique TMT reagent that served as a common reference for comparison to each patient sample across all four separate experiments. The pooled samples were then separated by offline pH reversed-phase liquid chromatography and analyzed by liquid chromatography-tandem MS (LC-MS/MS), using a hybrid quadrupole-Orbitrap mass spectrometer to generate RAW MS/MS data sets. As required, the four example RAW files were converted into MGF files for software compatibility, such as for SearchGUI/PeptideShaker searches in the Discovery module. In the writing, the abbreviations “RAW” and “MGF” were included to denote the file format of the input MS data sets, and in the figures, the input MS data sets were represented by the same RAW data set icons for simplification. As shown in Fig. 1, the Galaxy bioinformatics workflow consists of five modules (Database Generation, Discovery, Verification, Quantification, and Data Interpretation). All modules are summarized in Table S1, including details regarding inputs, outputs, and software tools. The complete workflow, data sets, and additional training resources are accessible via the Galaxy ecosystem and the GTN website (Tables S2 and S3).

### Database generation module for microbial peptide identification

To generate a comprehensive protein sequence database, a list of 118 taxonomic species that are commonly associated with the female reproductive tract was obtained from a 2018 metaproteomic study investigating the cervical-vaginal microbiome (64). In the 2018 study, a microbial database of 131 species was constructed using Human Microbiome Project reference genomes (64). For demonstration purposes in this study, the original list was shortened to 118 species, which consisted of 117 bacterial species and the yeast *Candida albicans* (Table S3).

Using this list of 118 taxonomic species, a protein sequence FASTA database (3,383,217 sequences) was generated using the UniProt XML Downloader tool within the Galaxy framework. Additionally, Human SwissProt (reviewed-only; 20,408 sequences, as of September 2023) and contaminant (cRAP; 116 sequences) protein sequence databases were generated using the Protein Database Downloader tool. The Species UniProt protein sequence database was then merged with the Human SwissProt (reviewed-only) and cRAP databases, using the FASTA Merge Files and Filter Unique Sequences tool to filter out duplicates and contaminants. This resulted in a comprehensive protein sequence database (2,595,745 sequences) (Fig. 2).

This comprehensive database, along with the four MS data sets (MGF) generated by LC-MS/MS analysis of PTF samples, were inputs for the MetaNovo tool to generate a reduced database (1,908 sequences) (12, 13). The MetaNovo tool infers proteins and organisms directly from raw MS data and the input protein sequence database to generate a reduced (targeted) database, without requiring exact prior knowledge of sample composition or metagenomic data generation (12). The MetaNovo tool has three components: DirecTag (for generating *de novo* sequence tags), PeptideMapper (for mapping sequence tags to a large FASTA sequence database), and an algorithm for probabilistic ranking of sequence database proteins and filtering based on estimated species and protein abundance (12). The resulting customized, targeted protein sequence database can be searched against raw MS data in PSM-based target-decoy analysis with greater sensitivity and FDR-controlled protein identification (12). The MetaNovo-generated database was then merged with the Human SwissProt (reviewed only) and cRAP databases to generate a compact database of 21,289 human and microbial sequences that were used for peptide identification.

### Discovery module using peptide identification programs

The four example MS data sets were searched against the compact database (21,289 sequences) to identify peptide sequences. Two peptide identification programs, SearchGUI/PeptideShaker and MaxQuant, were used for the searches (Fig. 3; Tables S4 and S5). For software compatibility, SearchGUI/PeptideShaker required the RAW files to be converted to MGF using the msconvert tool, whereas MaxQuant can process the RAW files.

SearchGUI is a database-searching tool that comprises different search engines to match sample MS/MS spectra to known peptide sequences (14, 18). In this analysis, the search algorithms MS-GF+ and X!Tandem within SearchGUI were employed to match spectra from MS data against peptides from the compact database (14, 15). Subsequently, PeptideShaker was used to organize the detected PSMs, assess the confidence of the data by using FDR analysis, and infer the identities of proteins based on the matched peptide sequences (18). Moreover, PeptideShaker generates outputs that can be used to visualize and interpret the data.

MaxQuant is an MS-based proteomics platform that is capable of processing raw data and provides improved mass precision and high precursor mass accuracy, which results in increased protein identification and more in-depth proteomic analysis (16, 17). Following database searching, microbial peptides from SearchGUI/PeptideShaker and MaxQuant were identified, merged, filtered to retain confident peptides, and grouped to obtain a list of distinct microbial peptides (Fig. 3). This list of distinct peptides was then extracted to use as input for PepQuery2 verification.

### Verification module of distinct microbial peptides using PepQuery2

Inputs for the PepQuery2 tool consisted of the list of distinct microbial peptides (from the Discovery module), the four example MS data sets (MGF), the Human UniProt Reference proteome (with isoforms; 82,678 sequences), and contaminants (cRAP) protein sequence databases. PepQuery2 tool further verified the identified microbial peptides detected via the Discovery module to ensure that they were indeed of microbial origin and not a result of human peptides being misassigned (Fig. 4A).

The PepQuery tool was developed as a peptide-centric search engine for MS/MS data analysis to verify the quality of non-host sequences by assigning *P*-values corresponding to the confidence level in peptide detection (19). PepQuery enables users to search peptide sequences against a proteomics database to verify high-confident PSMs and rigorously examines peptide modifications, which greatly reduces false discoveries in novel peptide identification (19). The PepQuery2 tool builds upon the capabilities of the initial PepQuery software release by providing a new MS/MS spectrum indexing, which results in highly efficient, targeted peptide identification (20). Parameters for PepQuery2 verification in this study are detailed in Table S6, and both versions of the tool are available as web-based and stand-alone applications (http://www.pepquery.org/).

For each MGF MS data set used as input, PepQuery2 generated a PSM rank file, each containing peptide identification information. These PSM rank files were compiled and filtered to retain confident PSMs. Then, the Query Tabular tool was used to select microbial peptides detected with confident PSMs and to remove potential contaminants (21). This list of microbial peptides was grouped to obtain distinct peptides (Fig. 4A). Then, as shown in Fig. 4B, PepQuery2-verified peptides and the peptides from SearchGUI/PeptideShaker and MaxQuant (from database searching from the Discovery module) were used as inputs for Query Tabular to extract accession numbers of proteins associated with the PepQuery2-verified peptides. The protein accession numbers of the verified microbial peptides were used to generate a protein sequences FASTA file using the UniProt XML Downloader tool within the Galaxy platform. This microbial protein sequences database (73 sequences) was then merged with the Human UniProt Reference proteome (with isoforms; 82,678 sequences) and cRAP sequence database to generate a protein sequences database (82,562 sequences) for protein and peptide quantification using MaxQuant software (Fig. 4B).

### Quantification module using MaxQuant

The Quantification module uses the verified microbial database from the previous module along with the Human UniProt and contaminants, RAW MS files, Experimental Design template (for MaxQuant), and Human protein sequences database as inputs (Fig. 5; Table S5). Proteins and peptides from the MS files were quantified using MaxQuant against the verified microbial protein sequences database (merged with human protein sequences and contaminants sequences; 82,562 protein sequences). The MaxQuant outputs of interest consist of a Protein Group text file, a Peptides text file, and an Evidence file. Within the Protein Group file, MaxQuant reported all proteins containing the sequences of identified peptides. For human proteins, wherein peptides are shared within isoforms, a group of proteins that share the peptides was reported. In microbial proteins, the protein groups are reported across organisms based on shared microbial peptides.

Since the inputs contained a mixture of human and microbial protein sequences and contaminants, MaxQuant outputs subsequently did not differentiate between microbial and human sequences. To perform data analysis of the microbial community present in the sample, microbial peptides were extracted from the list of identified peptides. This allowed us to prioritize the identification and quantification of microbial proteins, despite their lower abundance relative to the host proteins in the clinical samples. This module generated lists of quantified proteins and peptides that were processed for data interpretation and visualization (22).

### Data interpretation module using Unipept and MSstatsTMT

The Protein Group text file obtained through MaxQuant quantification was first divided into human and microbial protein groups. Proteins were designated with tags: “_HUMAN” for human host proteins and “_CON” for contaminant proteins. The remaining proteins with microbial taxonomy tags were designated as microbial proteins. To gain deeper insights into the microbial protein groups, the Unipept tool was employed to perform taxonomic and functional annotation. First, Unipept determined taxonomic composition by performing the Lowest Common Ancestor (LCA) analysis on the microbial peptide sequences (7, 23, 24). The Unipept tool has a variety of functions that allow for biodiversity analysis and data visualization of metaproteomics samples (23). The Unipept tool takes tryptic peptides as inputs, and for each given peptide, Unipept retrieves UniProt entries, which include accession numbers, protein names, and NCBI taxon IDs, from the UniProtKB database as well as a complete set of organisms in which the peptide occurs. Each peptide’s set of organisms is processed using the Unipept LCA algorithm to obtain the most specific taxonomic rank that the organisms share. For this study, the Unipept LCA analysis allowed for the assignment of the most likely genus, and species whenever possible, as well as functional categories for each of the 155 quantified microbial peptides (Fig. 6A).

Unipept outputs included a microbial taxonomic tree and enzyme commission proteins tree, hierarchical taxonomic annotation with EC numbers, InterPro, and Gene Ontology terms (Table S7). These outputs offered a comprehensive understanding of the microbial ecology of the quantified proteins.

Statistical analysis using MSstatsTMT relied on the Protein Group text file (.txt) obtained through MaxQuant quantification as well as the MaxQuant Evidence file (Fig. 6A; Tables S8 and S9). The human and microbial protein groups were separately analyzed using the MSstatsTMT tool. The MSstatsTMT tool is a free, open-source R/Bioconductor package that is compatible with data processing, such as MaxQuant, and allows for sensitive and specific detection of differentially expressed proteins in large-scale experiments with multiple biological samples (22, 25). MSstatsTMT, requiring annotation and comparison matrix files, removed all proteins labeled as contaminants from the MaxQuant protein groups and performed statistical analysis to discern the differential expression of quantified proteins across the three sample groups: healthy, benign, and OC. The annotation file dictated how the quantifications were combined, and the comparison matrix file was needed to accommodate the three different sample groups (OC, healthy, benign). MSstatsTMT generated tabular files with protein abundance ratios as well as comparison and volcano plots, all of which showcased differentially expressed proteins between human and microbial protein groups. Tutorial material for using MaxQuant and MSstatTMT for TMT data analysis is on the Galaxy Training Network (https://gxy.io/GTN:T00220).

## Data Availability

Input files can be accessed on Zenodo at https://doi.org/10.5281/zenodo.10720030 (last updated: February 2024). For future updates (if any), use the DOI 10.5281/zenodo.10105820 to access the most current version. All information for each module presented in this study is mentioned in the supplemental material, including example files and tools (and parameters) hosted on Galaxy Europe Server as well as Galaxy Training Network tutorials deposited on GitHub. All data and links are current as of February 2024.

## SUPPLEMENTAL MATERIAL

Supplemental Information msphere.00793-23-s0001.docx Supplemental figures and tables.

ASM does not own the copyrights to Supplemental Material that may be linked to, or accessed through, an article. The authors have granted ASM a non-exclusive, world-wide license to publish the Supplemental Material files. Please contact the corresponding author directly for reuse.
